# A randomized controlled pilot study of a brief web-based mindfulness training

**DOI:** 10.1186/1471-244X-11-175

**Published:** 2011-11-08

**Authors:** Tobias M Glück, Andreas Maercker

**Affiliations:** 1 University of Vienna, Faculty of Psychology, Institute of Clinical, Biological and Differential Psychology, Liebiggasse 5, A-1010 Vienna, Austria; 2 Division of Psychopathology and Clinical Intervention, University of Zurich, Binzmühlestr. 14/17, 8020 Zurich, Switzerland

## Abstract

**Background:**

Mindfulness has been shown to be effective in treating various medical and mental problems. Especially its incorporation in cognitive-behavioural interventions has improved long-term outcomes of those treatments. It has also been shown, that brief mindfulness-based trainings are effective in reducing distress. There have been few web-based interventions incorporating mindfulness techniques in their manual and it remains unclear whether a brief web-based mindfulness intervention is feasible.

**Methods:**

Out of 50 adults (different distress levels; exclusion criteria: < 18 years, indication of psychotic or suicidal ideation in screening) who were recruited via e-mail and screened online, 49 were randomized into an immediate 2-weeks-treatment group (N = 28) or a waitlist-control group (N = 21), starting with a 2-week delay. Distress (BSI), perceived stress (PSQ), mindfulness (FMI), as well as mood and emotion regulation (PANAS/SEK-27) were measured at pre-, post- and 3-month follow-up (3MFU). Intention-to-treat analyses using MI for missing data and per-protocol analyses (≥ 50% attendance) were performed.

**Results:**

26 participants of the treatment group completed post-measures. Most measures under ITT-analysis revealed no significant improvement for the treatment group, but trends with medium effect sizes for PSQ (*d *= 0.46) and PANAS^neg ^(*d *= 0.50) and a small, non-significant effect for FMI (*d *= 0.29). Per-protocol analyses for persons who participated over 50% of the time revealed significant treatment effects for PSQ (*d *= 0.72) and PANAS^neg ^(*d *= 0.77). Comparing higher distressed participants with lower distressed participants, highly distressed participants seemed to profit more of the training in terms of distress reduction (GSI, *d *= 0.85). Real change (RCI) occurred for PSQ in the treatment condition (OR = 9). Results also suggest that participants continued to benefit from the training at 3MFU.

**Conclusion:**

This study of a brief web-based mindfulness training indicates that mindfulness can be taught online and may improve distress, perceived stress and negative affect for regular users. Although there were no significant improvements, but trends, for most measures under ITT, feasibility of such a program was demonstrated and also that persons continued to use techniques of the training in daily life.

**Trial Registration:**

German Clinical Trials Register (DRKS): DRKS00003209

## Background

In recent years mindfulness has been found to be beneficial in various health related contexts [1,2]. It can be described as a form of mental training [3] where focus of attention is directed to present moment experiences in an open, curious and non-judgemental manner [4]. The technique to enter present moment experiences is usually the focus on breath or body sensations [5]. It is also important to note, that mindfulness is not restricted to formal meditation and can be incorporated in everyday activities [6]. It is however, not to be understood as a simple relaxation technique [7].

Most mindfulness trainings require participants to invest substantial amounts of time and discipline such as Mindfulness Based Stress Reduction (MBSR) [4]; however, short mindfulness-trainings lasting from a couple of days up to 4 weeks have also been reported effective in terms of mindfulness and distress reduction [8-12] and there is no evidence that shortened versions of mindfulness trainings are less effective [13].

Mindfulness has been described as *third wave *in cognitive-behavioural interventions [14] and is successfully incorporated in different cognitive-behavioural oriented treatments, e. g. for relapse prevention in depression [15]. With its incorporation into cognitive behavioural manuals, mindfulness is now also used in some web-based interventions as component in cognitive-behavioural treatment programmes for a variety of conditions [16-20]. Medium to large effect sizes have been reported in a programme for irritable bowel syndrome [18] and for depression [19]. Effects remained in a 6-month follow-up for the depression programme [19] and after 1 1/2 years for the irritable bowel syndrome study [21].

Generally for psychotherapeutic web-based treatments, medium effect sizes have been reported in meta-analyses on different web- and computer-based interventions [22-24]. Additionally, programmes with therapist contact seem to yield higher effect sizes than programmes that are self-guided [23,25]. However, to our knowledge there has been no study published focusing exclusively on the effectiveness and feasibility of a web-based mindfulness intervention. It remains unclear whether a programme exclusively consisting of mindfulness techniques is effective.

We wanted to evaluate whether a brief web-based mindfulness training could be delivered effectively via the internet for adults with different distress levels (ranging from lower to higher). We expected that the regular use of the training would have positive effects on distress and perceived stress, increase mindfulness, and improve emotion regulation and mood. We were also interested whether participants would continue to use the techniques, after the training had ended, and that beneficial effects on mindfulness and other measures would persist.

## Methods

### Participants

Participants were recruited in February 2010 over the internet by a short information e-mail containing a link to the official homepage at the University of Zurich. E-Mails were sent out to members of a students' club, faculty of both universities and employees of three companies (a car-dealership, a broadcasting station and a healthcare consulting company), asking to forward the information on the study to persons who might be interested in participating. They were also offered to participate themselves. These initially contacted persons (N = 98) in different occupational settings were chosen in order to reach a broader spectrum of educational levels and age-groups of potential participants. Approximately 400 persons received the information via e-mail.

**Figure 1:**
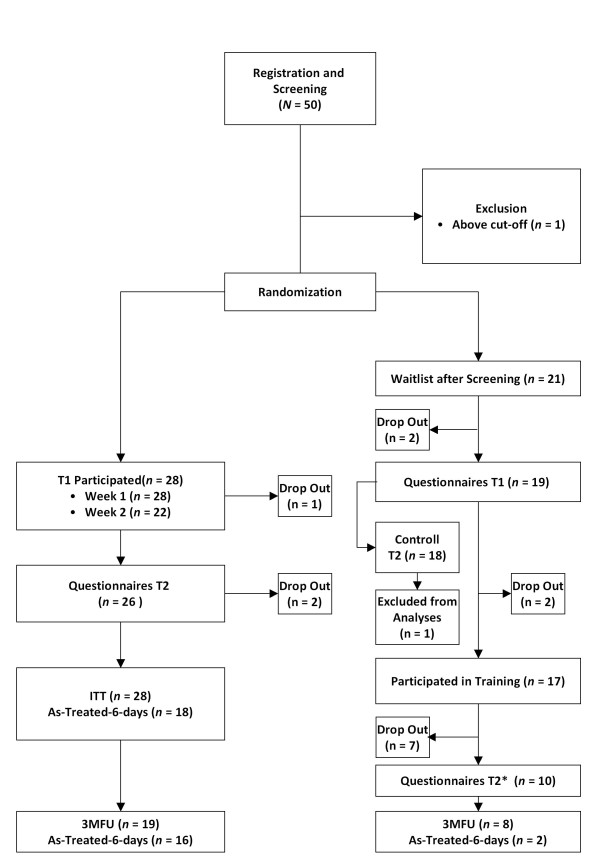
**Participant Flow Chart**.

On the information homepage persons could give consent to potentially participate and to be forwarded to the training's log-on and registration homepage, hosted by the University of Vienna. The study was conducted according to the ethical regulations for clinical trials of Austria and Switzerland. It was approved by the departments of psychology at both universities. 50 persons registered and completed the screening. One person exceeded cut-off in the screening and was excluded from the study before randomization, as depicted in Figure 1.

Persons aged under 18 years, or with indication of a psychotic disorder or suicidal ideation in the screening were excluded. Furthermore, persons were informed before they registered for the screening that they could not participate when they were currently pharmacologically or psychotherapeutically treated for a mental disorder or suffering of substance dependence. Persons indicating psychotic experience and/or suicidal ideation in the screening were contacted with information on counselling centres. Persons with higher distress (at least one of the nine screening scales exceeding a T-value of 63) were included in the study for later subgroup comparisons. They were informed, that they had indicated higher distress and were supplied with information on counselling centres as suggested in the manual [26].

Power was calculated using the software G*Power [27]. The power calculation was based on previous meta-analyses and individual research with a similar design on mindfulness interventions and distress reduction as well as effects of web-based interventions. Thus we expected a medium effect size between *d *= 0.50-0.70. With *p *≤ .05 (two-tailed) and power of a .80, in total 50 participants were required.

### Procedure

Using single-case randomization with previously created random number lists (assignment to even vs. uneven numbers), 49 persons received a standardized e-mail with information regarding their group-assignment within a day after they had completed the screening. We chose this approach to minimize information delay regarding the beginning date of the training. Due to our limited financial resources it was not possible to automatize the screening and randomization procedure within the program. However, this procedure resulted in unequal group sizes with 28 persons in the treatment condition and 21 persons in the waitlist-control group, who started with a two-week delay after the treatment group had finished the training.

**Figure 2:**
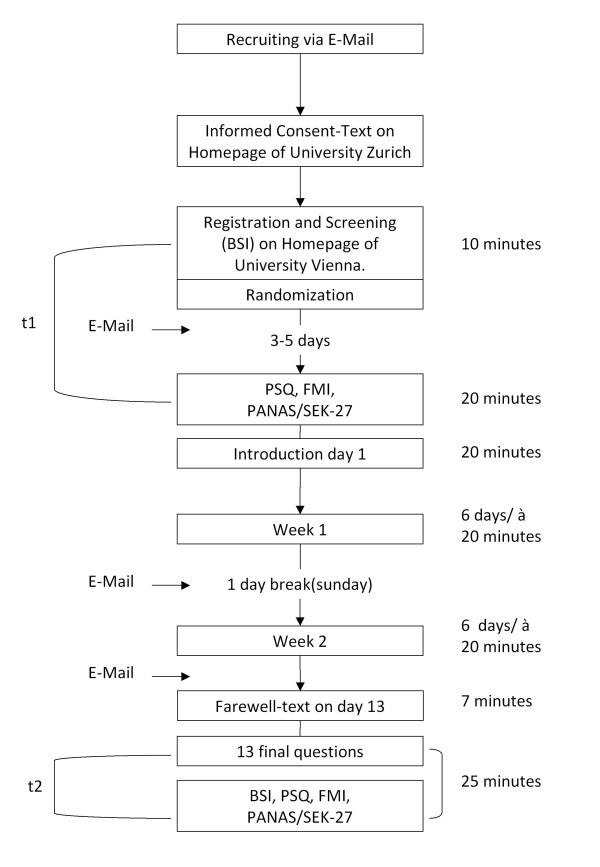
**Research design**.

Sociodemographic characteristics of groups are presented in Table 1. Participants in the treatment group were assessed at baseline and after the last session of the training. Follow-up at 3 month (3MFU) was completed by participants from both groups, as displayed in Figure 1. All questionnaires were completed online. The procedure of the training and time of measurement is depicted in Figure 2.

**Table 1:** Sociodemographic Characteristics

	Treatment (*n *= 28)	Waitlist (*n *= 21)
Age in Years *M *(*SD, Range*)	33.7 (12.7, 20-73)	37.2 (14.4, 22-68)
Female *n *(%)	20 (71.4)	16 (76.2)
Persons living in household	2	2
Education *n *(%)		
Secondary School	2 (7.1)	1 (4.8)
Grammar School	3 (10.7)	4 (19.0)
University Student	11 (39.3)	6 (28.6)
University Graduate	12 (42.9)	10 (47.6)
Meditationexperience *n *(%)		
none	10 (35.7)	5 (23.8)
little	13 (46.4)	9 (42.9)
some	3 (10.7)	5 (23.8)
much	2 (7.1)	2 (9.5)
Country *n *(%)		
Austria	15 (53.6)	11 (52.4)
Germany	7 (25.0)	6 (28.6)
Switzerland	6 (21.4)	4 (19.0)

### Measures

Internal consistencies in our sample matched those reported in the manuals of the instruments (Table 2).

**Table 2:** Internal consistencies of measures in sample

*Measure*	*BSI*	*PSQ*	*FMI*	*SEK-27*	*PANAS*
Cronbach α	.97	.87	.84	.89	.82

BSI measures distress. PSQ, perceived stress; FMI, mindfulness; SEK-27, emotion regulation; PANAS, positive and negative affect.

#### General distress

The global score (GSI) of the German *Brief Symptom Inventory *(BSI) [26], the 53 item version of the Symptom Checklist-90-R, was used to assess the general distress of participants.

#### Perceived stress

The subjective level of stress was assessed with the German 20-item version of the *Perceived Stress Questionnaire *(PSQ) [28]. The PSQ assesses the subjective level of stress on 4 dimensions: worries, tension, joy and demands. It also delivers a total score of the subjective stress level. It does not rely on a specific stressful situation. Items are rated on a scale from 1: *almost never*, 2: *sometimes*, 3: *often*, and 4: *usually*. The questionnaire was validated in a large German speaking sample consisting of different patient groups and healthy adults.

#### Mindfulness

To measure changes in mindfulness the German 14-item version of the *Freiburg Mindfulness Inventory *(FMI) was administered. Originally designed to measure changes of mindfulness during meditation retreats, it appears to be equally suitable for participants without meditation experience [29]. The short form shows good psychometric properties and items are rated on a scale from *Rarely*, *Occassionally*, *Fairly often*, to *Almost always *regarding their experiences with mindfulness. The short form is assumed to measure one general factor of mindfulness and thus a total score is calculated.

#### Emotion regulation and mood

To evaluate improvements in mood and different facets of emotion regulation skills the EMO-CHECK/SEK-27 [30] was used. It comprises two parts. The first part (EMO-CHECK) contains the 20 items of the *Positive Affect Schedule Negative Affect Schedule *(PANAS) [31] assessing two dimensions of negative affect and positive affect (further denoted as PANAS^neg ^and PANAS^pos^). In the second part (SEK-27) participants rate on 27 items (*never *to *almost always*) their competences of emotion regulation. Results may be interpreted on a general score.

#### Assessment of training perception

After each daily session participants answered 4 questions regarding their level of stress and how they had experienced that day's exercise. These questions were used to calculate the number of days they participated.

At the end of the training participants were asked 13 questions, how they liked the training, whether they were able to use the techniques and about design and usability. Questions were rated from -3 to 3 representing total disagreement and total agreement.

There were also 13 questions administered at the 3MFU which asked whether participants still practiced mindfulness exercises, when they used them and whether they were able to integrate mindfulness in their daily routine.

### Intervention

After randomization persons received an e-mail with general instructions and details on the training. The training always started on a Monday to ensure, that all participants would have equal conditions regarding weekdays. The training duration was 13 days and consisted of two modules. Each module lasted for 6 days with 20 minute-units per day. The modules were unlocked consecutively, and persons participated from Monday to Saturday.

All participants received standardized information and reminder-e-mails at the beginning of the training, after the first week (reminder for the second module), and at the end of the second module (reminder for post-test measures). Participants could also contact us via a contact-form on the homepage for technical assistance. Beyond that the training was self-guided without personal contact.

The training consisted of audio files, a flash animated exercise and written text. In the first module participants listened to an audio file with guided mindfulness exercises while being shown a neutral background-picture of pebbles on a white ground. Techniques included awareness of body sensations; attention to breath and acceptance of upcoming emotions [4,32]. In the second module participants were shown a blue sky. A cloud moved slowly across the sky, when pressing the spacebar once. They were instructed to practice the techniques learned in module 1 and when being disturbed by distressing thoughts, feelings or sensations, to label these cues non-judgementally (e.g. when feeling angry, to acknowledge it by simply labelling the internal image with "anger") and imagine placing them on the cloud, watching it wandering out of sight. Participants were instructed to press the key in full awareness, also being a marker to focus again on their breath or body sensations. This exercise was designed to support affect labelling and letting go, and was adapted from dialectic behavioural therapy [33].

### Statistical Analysis

Intent-to-treat analyses (ITT) were conducted on all participants who enrolled in the training and completed questionnaires at pre-test regardless of the number of days they used the training. All participants who filled in questionnaires at pre-test and dropped out in between were included using multiple imputation (MI) with 5 imputations for missing variables [34,35].

For per-protocol analyses only persons who participated on at least 6 days of the training and completed questionnaires at both times (pre- and post-test) were included in this analysis. This algorithm also assured that persons had participated in both weeks.

For pre-post-test changes, 2 × 2 repeated measures ANOVAs with time (pre-post-test) as within-subject variable and group as between-subjects variable were performed. For changes in the treatment group from post-test to follow-up, paired *t*-tests were computed. Effect sizes for main analyses are presented in Cohen's *d*. Effect sizes for paired *t*-tests were calculated with Dunlap et al.'s formula for Cohen's *d *[36]. Correlations (two-tailed) were also calculated with standardized response means [37] to analyze whether there were similarities to reported associations between FMI and other measures [29]. We also conducted mediation analysis according to Baron and Kenny [38] for daily exercise ratings for later outcome.

We calculated Reliable Change Indices (RCI) [39] to detect real changes in terms of improvement and deterioration. RCIs allow assessing whether a participant displays a real change with a probability of *p *≤ .05 when RCI-cut-off (± 1.96) is exceeded. Please note that this is only an indicator of real, but not necessarily clinically significant change [40]. Odds Ratios (OR) were calculated regarding favourable outcome in the treatment group. For 0-cells the conservative modified maximum likelihood estimate (MMLE) approach suggested by Gart and Zweifel [41-43] was calculated for ORs and confidence intervals.

All statistical analyses were computed at *p *≤ .05, two-tailed, using PASW Statistics 17 (SPSS Inc., Chicago).

## Results

### Attrition rate

Of the 49 persons who were eligible to participate, 44 (89.8%) filled in questionnaires at post-test. 27 persons (55.1%) also responded for the 3MFU. In the treatment group 64.3% (18 persons) participated for 6 or more days of the training over both weeks and 26 persons filled in questionnaires at post-test. 6 participants did not continue their practice in the second week of which two could not be reached for post-test. Participants with higher baseline levels of distress did not drop out more often than participants with lower baseline levels of distress. In the waitlist-group two persons dropped out after randomization and before pre-test (one person entered a correct, but inactive e-mail-address, the other person asked to be excluded for personal reasons). Participant flow is presented in Figure 1.

### Pre-treatment evaluation

Baseline differences for psychological parameters were analysed using independent group *t*-test, and showed statistically insignificant, but small to medium effect sizes for most measures. There was a significant difference between groups at baseline for positive affect with a medium effect size (Table 3). Levels of distress at pre-test in terms of T-Value means in both groups were statistically not different from population means described in the manual and a more recent validation study in a representative sample of the German population for the SCL-90-R [44]. It is also important to note, that irrespective of group, participants who completed grammar school (*F *(3, 43) = 3.83, *p *= .016), and those with little meditation experience had higher levels of distress (n.s., *F *(3, 43) = 2.27, *p *= .094) compared to other participants.

**Table 3:** Pre-treatment evaluation between treatment group and waitlist

*Measure*	*Treatment M(SD)*	*Waitlist M(SD)*	*t (df 45)*	*SE*	*p*	*d *[*CI %95*]
*GSI*	53.32 (16.14)	51.21 (10.83)	0.54	3.93	.594	0.16 [-0.42, 0.74]
*PSQ*	40.06 (16.39)	35.09 (13.40)	1.10	4.54	.261	0.33 [-0.26, 0.92]
*FMI*	37.04 (5.37)	39.95 (6.46)	-1.68	1.73	.100	-0.51 [-1.10, 0.08]
*SEK-27*	2.70 (0.48)	2.88 (0.56)	-1.22	0.15	.230	-037 [-0.96, -0.22]
*PANAS^neg^*	1.48 (0.93)	1.17 (0.65)	1.25	0.25	.218	0.38 [0.21, 0.97]
*PANAS^pos^*	2.54 (0.537)	2.86 (0.46)	-2.03	0.16	.048	-0.62 [-1.22, -0.02]

### Intent-to-treat analyses

Analysis suggested that data were missing completely at random (MCAR), *X^2 ^*_Little _= 30.52, *p *= .591.

#### Measures of distress—GSI and PSQ

PSQ showed a non-significant, but medium interaction effect, *F *(1, 45) = 2.64, *p *= .111, *d *= 0.46 [-0.13, 1.05], and a significant main effect for time, *F *(1, 45) = 4.19, *p *= .047. For GSI there was no significant interaction effect, *F *(1, 45) = 0.07, *p *= .794, *d *= 0.08 [-0.50, 0.66], and no significant main effect for time, *F *(1, 45) = 0.75, *p *= .391. Table 4 shows, that there was no further decrease in GSI, and a small, but non-significant effect for PSQ, *d *= -0.35, in the time after the training.

**Table 4:** Measures over time for treatment and waitlist control group

	Pre-test		Post-test		Follow-up		Treatment group, follow-up-post-test			
*Measure and group*	M	SD	M	SD	M	SD	*t *(*df *27)	SE	*p*	*d *[CI % 95]
*Distress (GSI)*										
Treatment	53.32	16.14	52.58	14.46	52.05	11.79	-.159	2.42	.874	-0.03[-0.37, 0.31]
Waitlist	51.21	10.83	49.67	10.63						
										
*Perceived Stress (PSQ)*										
Treatment	40.06	16.38	34.36	15.06	27.89	11.18	-1.638	3.14	.104	-0.35 [-0.78, 0.08]
Waitlist	35.09	13.39	34.72	15.35						
*Mindfulness (FMI)*										
Treatment	37.04	5.37	38.77	5.38	41.16	6.05	1.980	1.07	.054	0.32 [-0.01, 0.64]
Waitlist	39.95	6.46	40.67	6.78						
*Emotion Regulation (SEK-27)*										
Treatment	2.70	0.48	2.81	0.54	3.00	0.59	0.559	0.16	.580	0.12 [-0.31, 0.56]
Waitlist	2.88	0.56	2.98	0.49						
*Negative Affect (PANAS^neg^)*										
Treatment	1.48	0.93	0.96	0.70	0.97	0.48	0.158	0.16	.875	0.04 [-0.42, 0.49]
Waitlist	1.17	0.65	1.02	0.64						
*Positive Affect (PANAS^pos^)*										
Treatment	2.53	0.59	2.54	0.79	2.93	0.61	1.990	0.17	.049	0.43 [-0.01, 0.88]
Waitlist	2.86	0.46	2.89	0.54						

N = 47. Cohen's *d *for paired *t*-test calculated with Dunlap et al.'s formula [36]

#### Mindfulness—FMI

FMI showed a non-significant, but small effect for time-group interaction, *F *(1, 45) = 1.08, *p *= .304, *d *= 0.29 [-0.30, 0.88], and a significant main effect for time, *F *(1, 45) = 7.16, *p *= .010. Inspection of Table 4 shows a small effect for further increase in FMI from post-test to follow-up, *d *= 0.32.

#### Emotion regulation and mood—SEK-27 and PANAS

For SEK-27 there was no interaction, *F *(1, 45) = 0.02, *p *= .88, *d *= 0.05 [-0.53, 0.63], and no effect for time, *F *(1, 45) = 1.31, *p *= .258. PANAS^neg ^showed no significant time-group interaction, *F *(1, 45) = 3.69, *p *= .061, but with a medium effect, *d *= 0.50 [-0.09, 1.09], and a significant main effect for time, *F *(1, 45) = 14.24, *p *= .000. There was no significant effect for SEK-27 and PANAS^neg ^from post-test to follow-up (Table 4). PANAS^pos ^yielded no significant time-group interaction, *F *(1, 45) = 0.07, *p *= .794, *d *= 0.08 [-0.50, 0.66], and no main effect for time, *F *(1, 45) = 0.322, *p *= .573. However, there was a significant, medium effect for the time after the training to follow-up, *d *= 0.43 (Table 4).

### Per-protocol analyses

*n *was 18 for both groups. GSI showed no significant interaction, *F *(1, 34) = 0.54, *p *= .469, *d *= 0.29 [-0.30, 0.85], and no significant effect for time, *F *(1, 34) = 2.05, *p *= .162. PSQ yielded a significant interaction effect, *F *(1, 34) = 5.14, *p *= .030, *d *= 0.73 [0.13, 1.33], and a significant effect for time, *F *(1, 34) = 4.69, *p *= .037. FMI displayed a non-significant, but small interaction effect, *F *(1, 34) = 1.47, *p *= .234, *d *= 0.38 [-0.21, 0.97], and changed significantly over time, *F *(1, 34) = 6.41, *p *= .016. SEK-27 showed no interaction, *F *(1, 34) = 0.52, *p *= .478, *d *= 0.24 [-0.34, 0.82], and no significant main effect for time, *F *(1, 34) = 3.16. PANAS^neg ^showed a significant time-group interaction, *F *(1, 34) = 7.75, *p *= .009, with large effect, *d *= 0.77 [0.17, 1.37], and significant effect for time, *F *(1, 34) = 18.61, *p *= .000. PANAS^pos ^displayed trends with medium effects, but no significance for interaction, *F *(1, 34) = 2.84, *p *= .101, *d *= 0.56 [-0.03, 1.15], nor time, *F *(1, 34) = 2.61, *p *= .115.

### Ancillary Analyses

#### Subgroup analyses with highly distressed participants

This subgroup-analysis (*n *= 26) was conducted only with participants of the treatment group (pre-post-test). We compared participants with higher distress (*n *= 12, we defined higher distress as exceeding a T-value of 63 in any of the nine scales) against participants with lower levels of distress (*n *= 14) to see whether there was a difference in the effect of the training for persons with initial higher levels of distress. We computed 2 × 2 repeated measures ANOVAs. The groups did not differ in their initial FMI total score, *t *(26) = 0.52, *p *= .703, and there was also no difference in the amount of change of FMI between the two groups, *t *(24) = -0.39, *p *= .608. There was no significant time-group interaction effect for PANAS^neg ^*F *(1, 23) = 0.03 *p *= .877, *d *= 0, 05, and PSQ, *F *(1, 23) = 1.01, *p *= .325, *d *= 0.35. There was a significant and large time-group interaction effect for GSI, *F *(1, 23) = 4.56, *p *= .043, *d *= 0.85, and no significant main effect for time, *F *(1, 23) = 1.29, *p *= .268, *d *= 0.42. On average highly distressed individuals reduced their GSI score by 6 T-values (Table 5).

**Table 5:** Pre-test and post-test means for subgroup analysis

	Pre-test		Post-test	
***Measure and group***	**M**	**SD**	**M**	**SD**
*GSI*				
ND	41.43	7.10	43.21	7.78
HD	69.33	9.22	63.50	12.72
*PSQ*				
ND	33.09	17.17	28.69	12.46
HD	49.72	10.51	40.97	15.59
*FMI*				
ND	38.00	6.54	39.93	5.73
HD	36.00	3.52	37.42	4.81
*SEK-27*				
ND	2.71	0.51	2.95	0.58
HD	2.70	0.50	2.64	0.46
*PANAS^neg^*				
ND	1.22	0.91	0.66	0.34
HD	1.92	0.82	1.32	0.86
*PANAS^pos^*				
ND	2.62	0.58	2.62	0.71
HD	2.35	0.58	2.45	0.90

N = 26. ND = Normal distress (*n *= 14), HD = High distress (*n *= 12)

#### Individual indicators of change

To evaluate improvement and deterioration we calculated RCIs [39] for the different measures. For PSQ, 9 persons in the treatment condition showed significant real change versus 1 person in the waitlist group (*X*^2 ^= 5.12, *p *= .024, OR = 9.01). All other measures showed no significant difference between groups in terms of individual improvement (Table 6).

**Table 6:** Indication of real change pre-post-test (persons exceeding RCI-cutoff)

Improved				Deteriorated				
*Measure*	Treatment	Waitlist	*p*	OR [CI]	Treatment	Waitlist	*p)*	OR [CI %95]
*GSI*	7(29.9)	4(22.2)	1	1.29 [0.32, 5.28]	3 (11.5)	3(11.7)	.676	0.65 [0.12, 6.37]
*PSQ*	9(34.6)	1 (5.6)	.031	9.01 [1.03, 79.03]	0 (0.0)	2 (11.1)	.162	*0.12 [0.01, 2.70]
*FMI*	2 (7.7)	0 (0.0)	.505	*3.78 [0.17, 82.56]	0 (0.0)	0 (0.0)	1	*0.69 [0.01, 36.25]
*SEK-27*	3 (11.5)	2 (11.1)	1	1.04 [0.16, 6.97]	1 (3.8)	0 (0.0)	1	0.68 [0.04, 11.63]
*PANAS^neg^*	3 (11.5)	0 (0.0)	.258	*5.44 [0.26, 112.50]	0 (0.0)	0 (0.0)	1	*0.69 [0.01, 36.25]
*PANAS^pos^*	1 (3.8)	0 (0.0)	1	*2.14 [0.08, 55.76]	1 (3.8)	1 (5.6)	1	0.68 [0.04, 11.63]

N = 44. Numbers in () represent percentages within group. *p *calculated with Fisher's exact test. OR calculated for favourable outcome regarding treatment group. Numbers denoted with * represent estimated OR (MMLE).

#### Correlations of outcome measures and possible mediation

Negative coefficients for standardized scores of GSI, PSQ and PANAS^neg ^indicate improvement. For analysis data from treatment group and waitlist (after participating in the training) were combined (*N *= 35), and controlled for group. We found significant correlations between FMI and PSQ, *r *= -.68, *p *= .000, GSI, *r *= -.49, *p *= .004, and for PANAS^neg^, *r *= -.44, *p *= .009, for pre-post-test. For post-test to follow-up, association of FMI with GSI and PSQ remained, but not for PANAS^neg^, but there was also a strong association between FMI and SEK-27, *r *= .57, *p *= .003, which was negligible in pre-post-test. Mediation analysis [38] did not show any mediation effects of daily exercise ratings on post-test outcome; however, daily rating of engagement in exercise correlated with PANAS^pos^, *r *= .43, *p *= .009.

#### Subjective benefit and long-term use of training

Directly after the training 73.5%, and at the 3MFU 66.6% of the participants stated that they found the training to be beneficial and 70.3% had the feeling that the training had helped them regarding their inner balance and wellbeing. 45.7% of participants reported that the cloud used in the second week had helped them letting go. 77.2% would recommend the web-based mindfulness training. At 3MFU over 50% of the participants reported continued use of mindfulness techniques when they wanted to calm down in daily live. 25% reported to still regularly practice mindfulness exercises.

There were medium, but non-significant correlations between GSI and integration of exercises into daily routine, *r *= -.34, *p *= .101, and also for use of the cloud exercise from the second week to support letting go of negative or strong emotions (CLO), *r *= -.32, *p *= .124. There was a significant relationship between PANAS^neg ^and awareness of self and emotions, *r *= -.41, *p *= .045. We observed medium, but non-significant associations of PANAS^neg ^with CLO, *r *= -.31, *p *= .138, and for stating that the training had provided a good introduction to mindfulness techniques *r *= -.39, *p *= .057.

## Discussion

In this randomized controlled pilot study effects of a brief web-based mindfulness training on distress, perceived stress, mindfulness, mood and emotion regulation were investigated. Trends with medium effects in ITT and larger effects in per-protocol analysis suggest that a web-based mindfulness training may be effective in reducing perceived stress and improving negative affect.

In ITT we found medium, but non-significant effects for perceived stress (PSQ) and negative affect (PANAS^neg^). Interaction effects might have been influenced by baseline differences between groups, increasing overall variance. For mindfulness (FMI) and emotion regulation (SEK-27) there were non-significant, but small effects. Otherwise there were no trends or significant interaction effects in the ITT-analyses.

Despite methodological concerns [45], per-protocol analyses were performed, because we were also interested in the effects of the training when used regularly. The per-protocol analyses included persons who participated at least 50% of the training. This criterion was chosen, because it included persons who participated in both training modules. It has been postulated that only regular practice will result in changes of mindfulness [4]. This also corresponds with study results on neurobiological changes related to mindfulness exercise [46]. With persons participating at least for 6 days, the training showed to be effective for perceived stress (PSQ) and for negative affect (PANAS^neg^) with larger treatment effects, and trends with medium effects for positive affect (PANAS^pos^). Effects for PSQ are similar to stress reduction effects in a face-to-face mindfulness training in a community samples [47].

In most studies using the BSI as a measure of distress, significant changes are reported [48]; however, this was not the case in our study, with the exception of PSQ. In this respect it must be taken into consideration that the PSQ, which showed a medium effect in ITT and larger effect for per-protocol, is related to daily hassles and stress perception, while the BSI asks for symptom distress. Although there has been no evidence that shorter mindfulness trainings are less effective in reducing distress [13], it seems possible that subjective changes in distress were noticed only by those with higher initial levels of GSI. When conducting subgroup analyses for the treatment-group, participants with higher GSI at the beginning of the training reduced GSI scores more than participants with lower initial GSI. Higher distressed persons reduced on average by 6 T-values. It is also interesting to note, that current literature states that mindfulness techniques have been particularly helpful in distressed populations with medium to large effects for distress reduction [9].

Yet, it must also be kept in mind that persons with high levels of distress might be attracted by such trainings and thus expectations regarding the programme might account for some of the reduction in the distress score [49]. This pattern would also be expected for mindfulness outcome; on the other hand, participants with high distress levels did not increase their level of mindfulness in a different way than participants with lower levels of distress. Although mindfulness has been previously reported to mediate positive effects on psychological well-being [50] and perceived stress reduction [51], data in this study did not show these mediating effects, but there were some associations between the measures. Hence, we cautiously assume that reduction effects in maladaptive psychological parameters are related to positive changes in mindfulness. Furthermore we found expected correlations, and—if not significant—trends, between mindfulness (FMI) and the other measures. These correlations have also been reported in other studies [29,52-54].

In general, when analysing measures for real change as defined by Jacobson and Truax [39] we found that persons in the treatment condition were 9 times as likely to report a positive change in PSQ than participants in the control group. With the other measures we were not able to detect significant differences between the groups.

One of the purposes of the training was that persons would learn mindfulness techniques in a structured way to use them "offline" when the web-based training finished. This assumption was supported by small to medium effects for PSQ, *d *= -0.35, FMI, *d *= 0.32, and PANAS^pos ^*d *= 0.43, from post-test to follow-up.

Programme acceptance and satisfaction was high for this web-based training. Despite findings that self-guided web-based trainings and interventions seem to yield smaller effects than programmes in which therapists or instructors are included [22] this programme showed large effects on perceived stress for persons who regularly engaged in the training.

Although it is reported that attrition rates are a problem in web-based interventions [22] this was not the case for the treatment group between pre- and post-test. On the other hand, drop-out rates for the waiting-list group during training were far higher than in the treatment group, suggesting that the delay of training might have caused higher attrition rates in this group. This was contrary to findings of other studies incorporating a mindfulness component [19].

With the exercise introduced in module 2 a new form of interactive mindfulness exercise was tested. The exercise was inspired by Linehan's [33] cloud exercise, in which persons imagine to place distressing sensations or thoughts on a cloud, watching it passing by. It was taken care that this did not act as vigilance task paradigm [55], but as a support for the mindfulness aspect of letting go [56]. It was expected that this kind of affect labelling would mitigate emotional distress by supporting affect labelling in a non-judgmental manner. There were medium, but non-significant associations between a greater reduction of PANAS^neg ^and GSI with the use of the cloud technique in 3MFU. Also, when participants stated that they felt more aware of themselves and their emotions since they participated in the training, this was significantly associated with changes in PANAS^neg^. This is also supported by recent neurobiological studies on reduced amygdala response to emotional cues after affect labelling [57] also indicating that this mechanism is related to mindfulness exercise [58]. As the exercise was designed without spoken guidance, participants reported this to be more difficult.

### Limitations

The conclusions drawn from the results are limited by the heterogeneity of the data and also by group-differences in baseline measures with small to medium effect sizes. Graphical analysis of the data suggested, that this heterogeneity and baseline differences might have led to significance of some main time effects and non-significance of interaction effects in the analyses. A source of bias might also have been the recruitment of participants with different levels of initial distress, which could be responsible that interaction effects in GSI and FMI did not show significance. Another limitation was a missing control group in 3MFU, which should be addressed when conducting a larger scale study. Also the randomization procedure, using single-case randomization resulting in unequal sample sizes posed another limitation together with the limited power due to the small sample size.

Further limitations to generalizability are the high proportion of female participants, the high proportion of persons with academic background and the reliance on self-report. Especially education has been found to have a mediating influence on several aspects of mindfulness [50]. There has also been the claim, that mindfulness should be measured with other means but self-report measures for better validity [29]. As with most exercises and interventions offered online, it was not possible to control whether participants stayed in front of the screen and performed the exercises or did something different and simply returned after twenty minutes to log out. This however, will remain a problem with most web-based interventions [59].

## Conclusions

Although there were some limitations regarding the recruited sample and non-significant effects in ITT-analysis, this web-based, brief mindfulness training reduced negative affect and perceived stress for persons who participated at least in 50% of the training. It can also be assumed that a brief, web-based mindfulness programme may result in similar effects as face-to-face conducted mindfulness interventions [1], when used regularly. Furthermore, it may present an interesting adjunct to other web-based treatments.

To our knowledge there were only studies using mindfulness as a component within larger treatment protocols. It remained unclear, however, whether mindfulness did contribute to health improvements reported by these studies [18,19]. With this training we were able to show, that a brief mindfulness training is feasible over the internet and effective for some measures, when used regularly. On the other hand, there was only an indication, but no empirical evidence, that these effects were mediated by mindfulness.

For future research a better control of adherence and program use will be well advised. Future research should address the comparison of a web-based mindfulness programme compared to a web-based relaxation programme (e. g. progressive muscle relaxation or cognitive based stress reduction), as it has been conducted face-to-face [9,47]. In addition, treatment programmes with and without mindfulness components need to be compared to identify whether mindfulness is a beneficial adjunct for different web-based treatments. The results of this study are a first step in investigating the benefit of web-based mindfulness interventions.

## Competing interests

The authors declare that they have no competing interests.

## Authors' contributions

TMG wrote the manuscript, designed the training and conducted the statistical analysis. AM contributed to data analysis and writing the manuscript. AM also revised the first drafts and the final manuscript. Both authors developed the research design equally. Both authors approved the final version of the manuscript submitted for publication.

## Pre-publication history

The pre-publication history for this paper can be accessed here:

http://www.biomedcentral.com/1471-244X/11/175/prepub

## References

[B1] Baer RA Mindfulness training as a clinical intervention: A conceptual and empirical review Clin Psychol-Sci Pr 2003 10 2 125 143 10.1093/clipsy.bpg015

[B2] Hofmann SG Sawyer AT Witt AA Oh D The effect of mindfulness-based therapy on anxiety and depression: A meta-analytic review Journal of Consulting and Clinical Psychology 2010 78 2 169 183 20350028 10.1037/a0018555 PMC2848393

[B3] Bishop SR Lau M Shapiro S Carlson L Anderson ND Carmody J Segal ZV Abbey S Speca M Velting D Mindfulness: A proposed operational definition Clin Psychol-Sci Pr 2004 11 3 230 241 10.1093/clipsy.bph077

[B4] Kabat-Zinn J Full catastrophe living: Using the wisdom of your body and mind to face stress, pain and illness 1990 New York: Delacorte

[B5] Bishop SR What do we really know about mindfulness-based stress reduction? Psychosom Med 2002 64 1 71 83 11818588 10.1097/00006842-200201000-00010

[B6] Brown KW Ryan RM Perils and promise in defining and measuring mindfulness: Observations from experience Clin Psychol-Sci Pr 2004 11 3 242 248 10.1093/clipsy.bph078

[B7] Dimidjian S Linehan MM Defining an agenda for future research on the clinical application of mindfulness practice Clin Psychol-Sci Pr 2003 10 2 166 171 10.1093/clipsy.bpg019

[B8] Harnett P Whittingham K Puhakka E Hodges J Spry C Dob R The Short-Term Impact of a Brief Group-Based Mindfulness Therapy Program on Depression and Life Satisfaction Mindfulness 2010 1 3 183 188 10.1007/s12671-010-0024-3

[B9] Jain S Shapiro SL Swanick S Roesch SC Mills PJ Bell I Schwartz GE A randomized controlled trial of mindfulness meditation versus relaxation training: effects on distress, positive states of mind, rumination, and distraction Ann Behav Med 2007 33 1 11 21 10.1207/s15324796abm3301_2 17291166

[B10] Kingston J Chadwick P Meron D Skinner TC A pilot randomized control trial investigating the effect of mindfulness practice on pain tolerance, psychological well-being, and physiological activity J Psychosom Res 2007 62 3 297 300 10.1016/j.jpsychores.2006.10.007 17324679

[B11] Tang YY Ma YH Wang J Fan YX Feng SG Lu QL Yu QB Sui D Rothbart MK Fan M Short-term meditation training improves attention and self-regulation P Natl Acad Sci USA 2007 104 43 17152 17156 10.1073/pnas.0707678104 PMC2040428 17940025

[B12] Zeidan F Gordon NS Merchant J Goolkasian P The Effects of Brief Mindfulness Meditation Training on Experimentally Induced Pain J Pain 2010 11 3 199 209 10.1016/j.jpain.2009.07.015 19853530

[B13] Carmody J Baer RA How Long Does a Mindfulness-Based Stress Reduction Program Need to Be? A Review of Class Contact Hours and Effect Sizes for Psychological Distress Journal of Clinical Psychology 2009 65 6 627 638 10.1002/jclp.20555 19309694

[B14] Hayes SC Acceptance and commitment therapy, relational frame theory, and the third wave of behavioral and cognitive therapies Behav Ther 2004 35 4 639 665 10.1016/S0005-7894(04)80013-3 27993338

[B15] Teasdale JD Segal ZV Williams JMG Ridgeway VA Soulsby JM Lau MA Prevention of relapse/recurrence in major depression by mindfulness-based cognitive therapy Journal of Consulting and Clinical Psychology 2000 68 4 615 623 10965637 10.1037//0022-006x.68.4.615

[B16] Andersson G Kaldo V Internet-based cognitive behavioral therapy for tinnitus J Clin Psychol 2004 60 2 171 178 10.1002/jclp.10243 14724924

[B17] Eisen KP Allen GJ Bollash M Pescatello LS Stress management in the workplace: A comparison of a computer-based and an in-person stress-management intervention Comput Hum Behav 2008 24 2 486 496 10.1016/j.chb.2007.02.003

[B18] Ljotsson B Falk L Vesterlund AW Hedman E Lindfors P Ruck C Hursti T Andreewitch S Jansson L Lindefors N Internet-delivered exposure and mindfulness based therapy for irritable bowel syndrome—a randomized controlled trial Behav Res Ther 2010 48 6 531 539 10.1016/j.brat.2010.03.003 20362976

[B19] Meyer B Berger T Caspar F Beevers CG Andersson G Weiss M Effectiveness of a novel integrative online treatment for depression (Deprexis): randomized controlled trial J Med Internet Res 2009 11 2 e15 10.2196/jmir.1151 19632969 PMC2762808

[B20] Thompson NJ Walker ER Obolensky N Winning A Barmon C Diiorio C Compton MT Distance delivery of mindfulness-based cognitive therapy for depression: project UPLIFT Epilepsy Behav 2010 19 3 247 254 10.1016/j.yebeh.2010.07.031 20851055

[B21] Ljotsson B Hedman E Lindfors P Hursti T Lindefors N Andersson G Ruck C Long-term follow-up of internet-delivered exposure and mindfulness based treatment for irritable bowel syndrome Behav Res Ther 2011 49 1 58 61 10.1016/j.brat.2010.10.006 21092934

[B22] Andersson G Cuijpers P Internet-based and other computerized psychological treatments for adult depression: a meta-analysis Cogn Behav Ther 2009 38 4 196 205 10.1080/16506070903318960 20183695

[B23] Barak A Hen L Boniel-Nissim M Shapira Na A Comprehensive Review and a Meta-Analysis of the Effectiveness of Internet-Based Psychotherapeutic Interventions Journal of Technology in Human Services 2008 26 2/4 109 160

[B24] Preschl B Wagner B Forstmeier S Maercker A E-health interventions for depression, anxiety disorders, dementia, and other disorders in old age: A review Journal of CyberTherapy & Rehabilitation in press 21685658

[B25] Spek V Cuijpers P Nyklícek I Riper H Keyzer J Pop V Internet-based cognitive behaviour therapy for symptoms of depression and anxiety: a meta-analysis Psychological Medicine 2007 37 03 319 328 10.1017/S0033291706008944 17112400

[B26] Franke GH BSI. Brief Symptom Inventory - Deutsche Version. Manual 2000 Göttingen: Beltz Test GmbH

[B27] Faul F Erdfelder E Lang AG Buchner A G*Power 3: a flexible statistical power analysis program for the social, behavioral, and biomedical sciences Behav Res Methods 2007 39 2 175 191 10.3758/BF03193146 17695343

[B28] Fliege H Rose M Arck P Walter OB Kocalevent RD Weber C Klapp BF The Perceived Stress Questionnaire (PSQ) reconsidered: Validation and reference values from different clinical and healthy adult samples Psychosomatic Medicine 2005 67 1 78 88 10.1097/01.psy.0000151491.80178.78 15673628

[B29] Baer RA Smith GT Hopkins J Krietemeyer J Toney L Using self-report assessment methods to explore facets of mindfulness Assessment 2006 13 1 27 45 10.1177/1073191105283504 16443717

[B30] Berking M Znoj H Entwicklung und Validierung eines Fragebogens zu standardisierten Selbsteinschätzung emotionaler Kompetenzen (SEK-27) Zeitschrift für Psychiatrie, Psychologie, Psychotherapie 2008 56 2 1319349661

[B31] Crawford JR Henry JD The positive and negative affect schedule (PANAS): Construct validity, measurement properties and normative data in a large non-clinical sample Brit J Clin Psychol 2004 43 245 265 10.1348/0144665031752934 15333231

[B32] Hanh NT The Miracle of Mindfulness: An Introduction to the Practice of Meditation 1999 Boston, MA: Beacon Press

[B33] Linehan MM Skills training manual for treating borderline personality disorder 1993 New York: Guilford Press

[B34] Rubin DB Schenker N Multiple Imputation for Interval Estimation From Simple Random Samples With Ignorable Nonresponse Journal of the American Statistical Association 1986 81 394 366 374 10.2307/2289225

[B35] Schafer JL Multiple imputation: a primer Statistical Methods in Medical Research 1999 8 1 3 15 10.1191/096228099671525676 10347857

[B36] Dunlap WP Cortina JM Vaslow JB Burke MJ Meta-analysis of experiments with matched groups or repeated measures designs Psychological Methods 1996 1 2 170 177

[B37] Stratford PW Binkley FM Riddle DL Health status measures: strategies and analytic methods for assessing change scores Phys Ther 1996 76 10 1109 1123 8863764 10.1093/ptj/76.10.1109

[B38] Baron RM Kenny DA The moderator-mediator variable distinction in social psychological research: conceptual, strategic, and statistical considerations J Pers Soc Psychol 1986 51 6 1173 1182 3806354 10.1037//0022-3514.51.6.1173

[B39] Jacobson NS Truax P Clinical significance: a statistical approach to defining meaningful change in psychotherapy research J Consult Clin Psychol 1991 59 1 12 19 2002127 10.1037//0022-006x.59.1.12

[B40] Jacobson NS Roberts LJ Berns SB McGlinchey JB Methods for defining and determining the clinical significance of treatment effects: description, application, and alternatives J Consult Clin Psychol 1999 67 3 300 307 10369050 10.1037//0022-006x.67.3.300

[B41] Parzen M Lipsitz S Ibrahim J Klar N An Estimate of the Odds Ratio That Always Exists Journal of Computational and Graphical Statistics 2002 11 2 420 436 10.1198/106186002760180590

[B42] Agresti A On Logit Confidence Intervals for the Odds Ratio with Small Samples Biometrics 1999 55 2 597 602 10.1111/j.0006-341X.1999.00597.x 11318220

[B43] Gart JJ Zweifel JR On the Bias of Various Estimators of the Logit and Its Variance with Application to Quantal Bioassay Biometrika 1967 54 1/2 181 187 10.2307/2333861 6049534

[B44] Hessel A Schumacher J Geyer M Brähler E Symptom-Checklist SCL-90-R Diagnostica 2001 47 1 27 39 10.1026//0012-1924.47.1.27

[B45] Ellenberg JH Intent-to-treat analysis versus as-treated analysis Drug Information Journal 1996 30 10

[B46] Davidson RJ Kabat-Zinn J Schumacher J Rosenkranz M Muller D Santorelli SF Urbanowski F Harrington A Bonus K Sheridan JF Alterations in brain and immune function produced by mindfulness meditation Psychosomatic Medicine 2003 65 4 564 570 10.1097/01.PSY.0000077505.67574.E3 12883106

[B47] Smith BW Shelley BM Dalen J Wiggins K Tooley E Bernard J A pilot study comparing the effects of mindfulness-based and cognitive-behavioral stress reduction J Altern Complement Med 2008 14 3 251 258 10.1089/acm.2007.0641 18370583

[B48] Shapiro SL Astin JA Bishop SR Cordova M Mindfulness-Based Stress Reduction for Health Care Professionals: Results From a Randomized Trial International Journal of Stress Management 2005 12 2 164 176

[B49] Greenberg RP Constantino MJ Bruce N Are patient expectations still relevant for psychotherapy process and outcome? Clin Psychol Rev 2006 26 6 657 678 10.1016/j.cpr.2005.03.002 15908088

[B50] Baer RA Smith GT Lykins E Button D Krietemeyer J Sauer S Walsh E Duggan D Williams JM Construct validity of the five facet mindfulness questionnaire in meditating and nonmeditating samples Assessment 2008 15 3 329 342 10.1177/1073191107313003 18310597

[B51] Carmody J Baer RA Relationships between mindfulness practice and levels of mindfulness, medical and psychological symptoms and well-being in a mindfulness-based stress reduction program J Behav Med 2008 31 1 23 33 10.1007/s10865-007-9130-7 17899351

[B52] Brown KW Ryan RM The benefits of being present: mindfulness and its role in psychological well-being J Pers Soc Psychol 2003 84 4 822 848 12703651 10.1037/0022-3514.84.4.822

[B53] Cardaciotto L Herbert JD Forman EM Moitra E Farrow V The assessment of present-moment awareness and acceptance: the Philadelphia Mindfulness Scale Assessment 2008 15 2 204 223 10.1177/1073191107311467 18187399

[B54] Baer RA Smith GT Allen KB Assessment of mindfulness by self-report: the Kentucky inventory of mindfulness skills Assessment 2004 11 3 191 206 10.1177/1073191104268029 15358875

[B55] Robertson IH Manly T Andrade J Baddeley BT Yiend J 'Oops!': performance correlates of everyday attentional failures in traumatic brain injured and normal subjects Neuropsychologia 1997 35 6 747 758 10.1016/S0028-3932(97)00015-8 9204482

[B56] Kabat-Zinn J Mindfulness-based interventions in context: Past, present, and future Clin Psychol-Sci Pr 2003 10 2 144 156 10.1093/clipsy.bpg016

[B57] Lieberman MD Eisenberger NI Crockett MJ Tom SM Pfeifer JH Way BM Putting feelings into words - Affect labeling disrupts amygdala activity in response to affective stimuli Psychol Sci 2007 18 5 421 428 10.1111/j.1467-9280.2007.01916.x 17576282

[B58] Creswell JD Way BM Eisenberger NI Lieberman MD Neural correlates of dispositional mindfulness during affect labeling Psychosomatic Medicine 2007 69 6 560 565 10.1097/PSY.0b013e3180f6171f 17634566

[B59] Danaher BG Seeley JR Methodological Issues in Research on Web-Based Behavioral Interventions Ann Behav Med 2009 38 1 28 39 10.1007/s12160-009-9129-0 19806416 PMC3846298

